# Case report of a delayed iatrogenic Pipkin type III femoral head fracture-dislocation

**DOI:** 10.1097/MD.0000000000028773

**Published:** 2022-01-28

**Authors:** Qin-Wen Li, Cai-Sheng Zhou, Yu-Peng Li

**Affiliations:** Department of Orthopedics, The People's Hospital of Three Gorges University, The First People's Hospital of Yichang, Yichang, Hubei, China.

**Keywords:** case report, iatrogenic, irreducibility, Pipkin III fracture

## Abstract

**Rationale:**

Pipkin III femoral head fracture dislocation (FHFD) is rarely observed in clinical practice, and its outcome is alarming. A considerable proportion of Pipkin III fractures result from repeated or forceful closed reduction of an irreducible FHFD. Pipkin type III fractures pose a therapeutic challenge. Most patients underwent total hip arthroplasty.

**Patient concerns:**

A 34-year-old man experienced high-energy trauma to the left hip from a terrible traffic accident. Radiography and computed tomography (CT) at the local hospital revealed a left posterior FHFD. Emergency close reduction of the hip was performed.48 hours later, the patient was transferred to our institution. New radiography and CT examinations demonstrated an iatrogenic femoral neck fracture (FNF) associated with FHFD. In addition, a right talar fracture was observed.

**Diagnosis:**

Pipkin III fracture combined with contralateral talar fracture.

**Interventions:**

Considering his Pipkin fracture classification (Pipkin Type-III) and the time to surgery after his injury (>48 hours), the patient underwent left total hip arthroplasty uneventfully, followed by below-ankle plaster cast immobilization for his right ankle.

**Outcomes:**

At the 6-month follow-up, the patient was able to walk pain-free, and plain radiographs were satisfactory, with no evidence of heterotopic ossification or osteonecrosis of the talus.

**Lessons:**

Before emergency closed reduction, early recognition of the unique characteristics of an irreducible FHFD is essential to avoid iatrogenic femoral neck fracture.

## 1 Introduction

In 1869, Birkett first discovered and documented a fracture of the femoral head during cadaver dissection.^[1]^ Approximately 93% of such fractures typically result from motor vehicle accidents with high impact, occurring in the case of polytrauma.^[2]^ These high-energy fractures are infrequent, accounting for approximately 5% to 15% of all posterior hip dislocations.^[3]^ According to the classification system, Pipkin type III injuries constitute a subgroup of femoral head fractures associated with ipsilateral femoral neck fractures (FNFs).^[4]^ As the least common 1, Pipkin Type-III injuries contributed to 8.6% of all the Pipkin fractures. Restricted by the complexity of the reconstruction of the natural hip joint, Pipkin type III fractures presented a worse prognosis than Pipkin type I or II fractures. It is worth noting that none of the type-III fractures had a good prognosis.^[5]^ Pipkin type III fractures pose challenges in disease management. Major complications of posttraumatic osteoarthritis, avascular necrosis (AVN), and heterotopic ossification (HO) may result in restricted hip function and permanent disability in young patients.^[6]^ Here, we report a case of delayed iatrogenic Pipkin type III femoral head fracture dislocation (FHFD) with high-energy trauma. We illustrated the unusual mechanisms underlying iatrogenic Pipkin type III fractures and provided recommendations for treating such fractures based on a literature review.

## 2 Consent

The current study was approved by the ethics committee of the People's Hospital of Three Gorges University/The First People's Hospital of Yichang. Patient consent was obtained.

## 3 Case report

**Figure 1:**
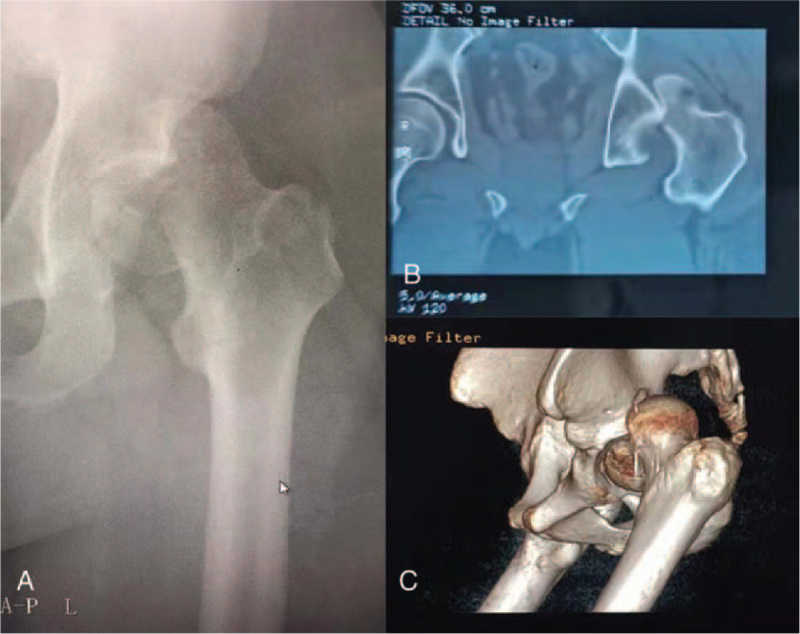
X-ray as well as computed tomography (CT) examination before closed reduction of hip dislocation. (A) Anteroposterior x-ray image of the left hip. (B) Coronal CT scan showing femoral head fractures and dislocation of hip joint. (C) 3-Dimensional CT of hip showing femoral head fractures and posterior dislocation of hip joint.

**Figure 2:**
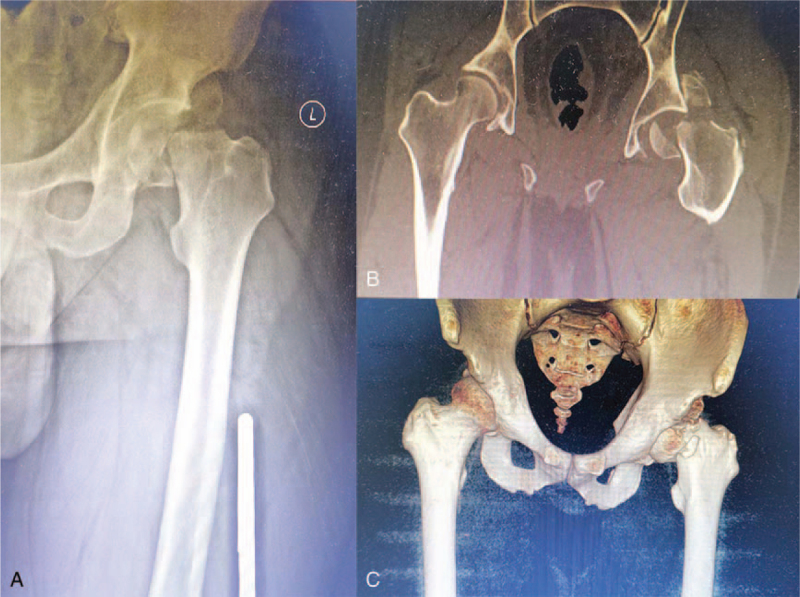
New X-ray and CT examination after closed reduction of hip dislocation. (A) Anteroposterior radiograph of the left hip. (B) Coronal CT images showing femoral head and femoral neck fractures. (C) 3-Dimensional CT images of hip showing femoral head and femoral neck fractures with fragments of the femoral head fracture remained in the acetabular fossa.

**Figure 3:**
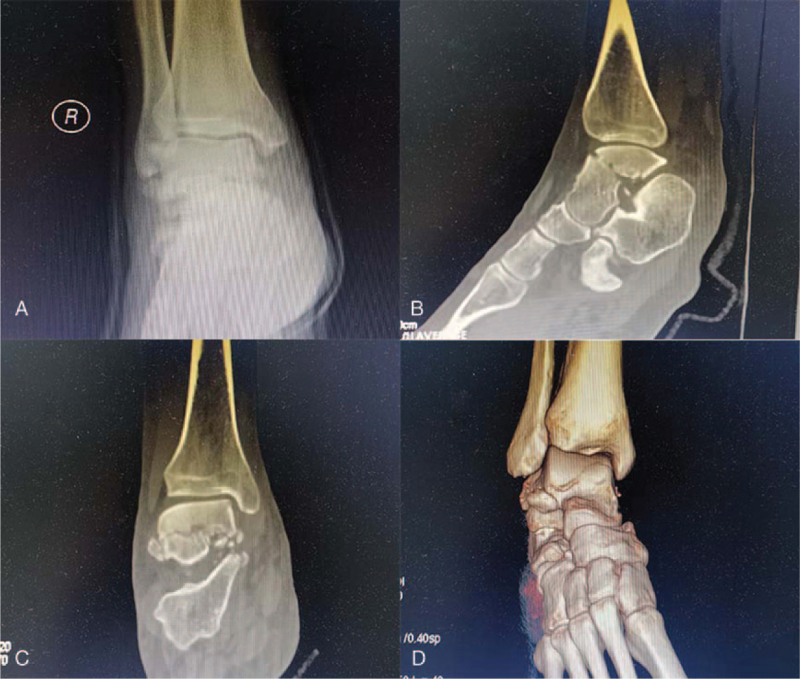
X-ray and CT examination showing right talus fracture. (A) Anterior-posterior view. (B) Sagittal image of CT.(C) Coronal image of CT. (D) 3-Dimensional reconstruction of CT.

A 34-year-old man experienced traumatic injury to the left hip following a terrible traffic accident. The patient was sent to the local hospital quickly and underwent radiography along with computed tomography (CT), and the result demonstrated a left posterior FHFD (Fig. 1). Due to severe dislocation along with the fracture, the posterior margin of the acetabulum blocked the femoral head or neck. Unfortunately, doctors in the local hospital were unaware of its irreducibility and performed emergency close reduction of hip dislocation for the patient, suggesting that close reduction had reduced the dislocated hip without giving any radiographing thereafter. Subsequently, femoral supracondylar traction was performed. Two days later, the patient was transferred to our institution for improved treatment and nursing. It has been over 48 hours since the injury occurred when he arrived at our institution. New radiographs and CT examinations were performed to assess fracture–dislocation of the hip. In addition to an iatrogenic FNF associated with FHFD (Fig. 2), new radiographs and CT examinations also revealed a right talar fracture (Fig. 3).

**Figure 4:**
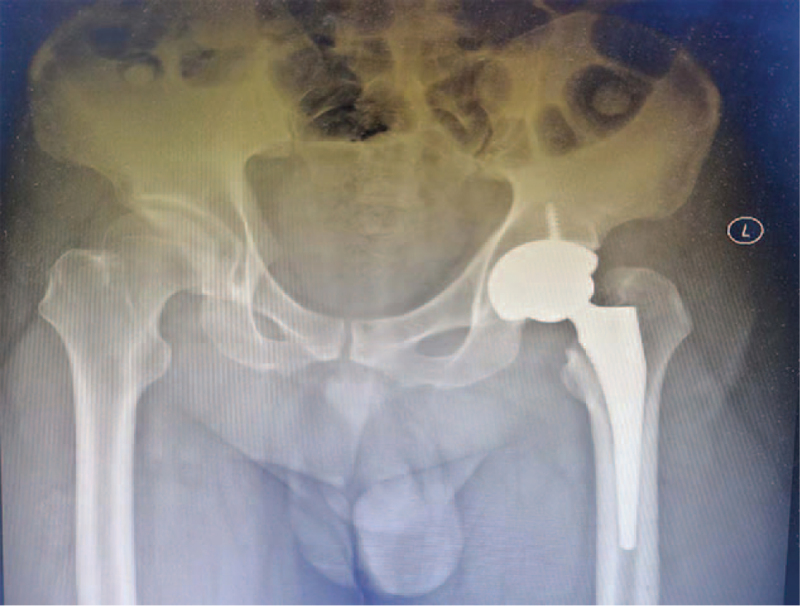
Pelvic plain radiograph showing postleft total hip arthroplasty.

Considering his Pipkin fracture classification (Pipkin Type III) and the time to surgery after his injury (>48 hours), we counseled the patient for left total hip arthroplasty (THA) rather than open reduction and internal fixation (ORIF). The patient underwent left THA uneventfully (Fig. 4). Considering the right talar fracture, his right ankle was protected with below-ankle plaster cast immobilization and nonweight-bearing for 6 weeks. After the operation, rehabilitation was performed as soon as possible according to our instructions. During the early postoperative stage, the patient performed active hip flexion and extension exercises on the bed. Early weight bearing was also allowed for the left hip. After the surgery, he resumed his work duty as a driver for 3 months. Follow-up examinations were conducted 6 months later, and plain radiographs were satisfactory with no evidence of HO. The patient could also walk without feeling any pain. Clinical evaluation of the hip joint using the Harris hip scale (HHS = 92) indicated that his outcome was excellent. At follow-up, the patient had no clinical or radiological features suggestive of talar osteonecrosis.

## 4 Discussion

Compared to other hip fractures, FHFD is uncommon in clinical practice. Pipkin classified FHFD into 4 subtypes, among which type III is the least frequently reported in the literature, representing catastrophic injuries.^[7]^ Fractures of the femoral head and neck imply a dual insult to the proximal femur, which is an urgent situation in orthopedic trauma. Some Pipkin type III fractures result from severe or high-energy trauma to the hip or lower extremity, whereas others may have been caused by repeated or forceful closed reduction of irreducible FHFD injuries.^[8]^ As reported in the literature, the irreducibility of FHFD has not been recognized to a great extent, leaving iatrogenic Pipkin type-III fractures as a major concern.^[9]^ In our case, the local doctors attempted emergent closed reduction without increasing the risk of irreducible FHFD. Finally, iatrogenic FNF was identified through three-dimensional (3D) reconstructed CT images once the patient arrived at our institution.

Successful closed reduction may not always be feasible in FHFD. The unique features defining the irreducibility of FHFD injuries may include the injured lower limb positioned at the hip joint in slight but fixed flexion accompanied by immobile neutral rotation along with obvious anisomelia, which should be acquired by both physicians in emergency departments and orthopedic surgeons, preventing further FNF with resultant iatrogenic Pipkin type III injuries.^[10]^ Owing to its rarity, it is impossible to study iatrogenic Pipkin type III fractures in large patient populations. Thus, researchers can use retrospective imaging data to determine the particular characteristics of irreducible hip dislocations. After careful examination of X-ray and CT scans of irreducible cases, a special finding was revealed from the views of the obturator foramen: the close apposition of the femoral head-neck-shaft component against the lateral iliac cortical bone of the supra-acetabular region.^[8,11]^ These viewpoints of imaging studies may be significant for the identification of irreducible FHFD cases. Thus, different therapeutic strategies to avoid iatrogenic FNF are suggested, rather than attempts at closed reduction in cases of irreducibility. In this case, the initial CT images revealed that the fractured femoral head perched on the sharp angle of the posterior wall of the acetabulum, which was consistent with the irreducibility of posterior dislocation of the hip. Therefore, before attempting closed reduction, careful physical examination and early recognition of radiographic hallmarks are essential to determine the most appropriate treatment strategy, while avoiding related complications.

As Pipkin type III fractures are uncommon, data analyzing the outcomes of these fractures are currently lacking. Traditionally, preservation, along with the restoration of native hip biomechanics through ORIF, has been the treatment goal. So far, there has been occasional reports on effective ORIF without complication of AVN of the femoral head.^[12,13]^ However, most outcomes of ORIF management have been reported to be frustrating. In a prospective study, 7 type-III fractures were identified in 147 Pipkin injuries over a period of 13 years.^[14]^ ORIF was performed in all cases. However, all Pipkin III fractures failed to undergo operative fixation and required conversion to THA. Similarly, in a retrospective study of 110 Pipkin fracture charts, 4 Pipkin III fractures were identified, among which one was treated with first-intention THA, while the remaining 3 finally underwent secondary THA because of the failure of initial treatments.^[15]^ Pipkin III injuries are also characterized as “intra-articular segmental proximal femoral fractures.” The displaced FNFs compromise the vascular supply of the femoral head by means of increased intracapsular pressure, direct disruption and kinking of the veins, and subsequent decrease in head perfusion.^[16]^ Moreover, prolonged dislocation of the hip joint accelerates the lack of blood supply to the femoral head, which may result in AVN of the femoral head.^[17]^ The remaining blood supply to the femoral head may be better preserved with early and successful reduction. Prompt reduction should be performed as soon as possible, preferably within 6 hours, to decrease the risk of femoral head osteonecrosis.^[18]^ A recent report showed that Pipkin injuries with restoration of the articular surface earlier than 48 hours showed better outcomes.^[19]^ Owing to the damaged vascular supply to the concomitant femoral head, ORIF is more challenging. The posterolateral (Kocher-Langenbeck) and anterior (Smith-Petersen) approaches are the most commonly used approaches in the literature. The Kocher-Langenbeck approach can easily damage the deep branch of the medial femoral circumflex artery (MFCA), which is the main source of the femoral head vascular supply. It also interrupts the anastomosis between the deep branch of the MFCA and inferior gluteal artery. Comparatively, patients treated with the Smith-Petersen approach have been found to develop a more significant incidence of HO but showed no influence on MCFA.^[20,21]^ Over the past few years, modified ORIF approaches have gained considerable popularity. However, there has been no consensus on the management of Pipkin III fractures regarding the operative approach.

However, given the challenges of ORIF, THA seems to offer an alternative option. Previous studies have provided conservative views regarding THA in young individuals. These studies mainly utilized first-generation implants; comparatively, the use of new modern implants and improved surgical techniques have led to better outcomes. With the continuous evolution of the preferred bearing surface for young patients, the ideal bearing surface is identified for extremely young patients undergoing primary THA to improve survivability and reduce complications of the bearing surface of THA.^[22]^ It is well documented that a large amount of evidence has proven good outcomes of THA among young patients with end-stage hip diseases attributed to nontraumatic factors, spurring recommendations for primary THA for type III fractures.^[23]^ Swarup et al.^[24]^ reported that 135 young patients (mean age, 27 years) with femoral head necrosis underwent primary THA and presented with a good implant survival rate and favorable long-term follow-up results. These findings suggest that young patients might be capable of functioning very well after THA. Based on the above evidence, as well as our clinical experience, the authors suggest that THA is preferable for our case rather than challenging ORIF.

## 5 Conclusion

We report a case of delayed iatrogenic Pipkin III fractures combined with a contralateral talar fracture in a 34-year-old man. Guidelines on proper management are lacking in the literature. Due to challenging ORIF and various factors, our patient underwent primary THA and below-ankle plaster cast immobilization, resulting in satisfactory implant survival and excellent hip functional outcome at the 6-months follow-up. Irreducible FHFD can be predicted based on specific clinical and radiographic features. For surgeons in charge of the emergent assessment and treatment of questionable cases, special consideration of irreducible FHFD injury patterns is warranted. Hasty or repeated closed reductions should be avoided. Instead, open reduction is recommended for such injuries to avoid iatrogenic Pipkin type III fractures.

## Author contributions

**Conceptualization:** Qinwen Li.

**Data curation:** Qinwen Li.

**Formal analysis:** Qinwen Li.

**Investigation:** Cai-Sheng Zhou.

**Methodology:** Cai-Sheng Zhou.

**Project administration:** Yu-Peng Li.

**Resources:** Cai-Sheng Zhou.

**Software:** Cai-Sheng Zhou.

**Supervision:** Yu-Peng Li.

**Validation:** Yu-Peng Li.

**Visualization:** Yu-Peng Li.

**Writing – original draft:** Qinwen Li.

**Writing – review & editing:** Qinwen Li.
